# Acute exercise and mindfulness meditation on learning and memory: randomized controlled intervention

**DOI:** 10.15171/hpp.2019.43

**Published:** 2019-10-24

**Authors:** Malina Austin, Paul D. Loprinzi

**Affiliations:** Exercise & Memory Laboratory, Department of Health, Exercise Science and Recreation Management, The University of Mississippi, University, MS 38677

**Keywords:** Awareness, Cognition, Exercise, Meta-cognition

## Abstract

**Background:** The purpose of this experiment was to evaluate the potential combined effects of acute exercise and mindfulness mediation on episodic memory.

**Methods:** All data collection occurred in the authors’ laboratory (January to May of 2019). In this three-arm, within-subject design, participants (N=20; M_age_=21.6 years) completed three counterbalanced laboratory visits, including Exercise Only, Exercise + Meditation and Control. Learning and memory were assessed from a word-list task. A one-factor repeated-measures ANOVA was computed for two memory outcomes, including the learning outcome (average performance across the 6 trials) and the long-term memory recall (10-minute delay).

**Results:** The exercise conditions had a greater learning effect when compared to the Control visit, M_diff_ = 0.68 (95% CI: 0.10, 1.25), P = 0.02. The Exercise + Memory visit had better longterm memory when compared to Exercise Only, M_diff_ = 0.95 (95% CI: 0.07, 1.83), P = 0.03.

**Conclusion:** The present experiment provides suggestive evidence that acute exercise may enhance learning and, when coupling acute exercise prior to encoding with meditation during early consolidation, long-term memory may be enhanced.

## Introduction

Among young adults, emerging experimental work has started to examine the effects of exercise on memory function, providing suggestive evidence that acute exercise may enhance learning and memory function.^1-4^ We have previously detailed the dearth of research on this topic among this population.^5^ The mechanisms through which exercise may influence learning and episodic memory function has also been extensively detailed by our group.^6^ Such mechanisms include, for example, exercise-induced alterations in growth factors (e.g., brain-derived neurotrophic growth factor, insulin-like growth factor-1),^7-9^ ultimately facilitating long-term potentiation,^10^ or the functional connectivity among neurons.

In addition to acute exercise, emerging work suggests that mindfulness meditation may also play an important role in subserving cognition. For example, we recently demonstrated that a combination of acute exercise and mindfulness meditation was optimal in enhancing aspects of executive function,^11^ which has been shown to influence memory function.^12^ Further, after memory encoding, mindfulness meditation has been shown to facilitate motor memory consolidation.^13^

Also, as recommended elsewhere,^5^ no experimentation has examined the potential individual and combined effects of acute exercise and meditation on memory, which was the purpose of this study. Such a combined effect is plausible as both exercise and meditation have been shown to enhance aspects of memory formation. Given the potential memory consolidation effects of meditation, coupled with the potential overall learning and memory effects of acute exercise, for this experiment, we strategically placed the acute bout of exercise prior to memory encoding and the acute meditation session post-memory encoding. We hypothesized that acute exercise by itself would facilitate learning and memory, whereas a condition of acute exercise (prior to encoding) and acute meditation (post-encoding) would be optimal for long-term memory function.

## Materials and Methods

### Study design

All data collection occurred in the authors’ laboratory (January to May of 2019) at the University of Mississippi. In this three-arm, within-subject design, participants completed three counterbalanced laboratory visits, including Exercise Only, Exercise + Meditation and Control. The Exercise Only visit engaged in an acute bout of exercise prior to a learning task. The Exercise + Meditation visit engaged in an acute bout of exercise prior to learning, and then during the memory consolidation stage, they engaged in a brief mindfulness meditation session. Lastly, the Control visit did not involve any acute exercise or mindfulness meditation. All visits occurred around the same time of day and were separated by approximately 24-48 hours. This study was approved by the ethics committee at the University of Mississippi (#18-115, approved on May 30, 2018). Participants provided written consent prior to participation. Schematic of the study procedures is shown in Table 1.

### Participants

In total, 20 participants completed all three visits. This aligns with other related experimental work.^1-4^ Recruitment occurred via a convenience-based, non-probability sampling approach (classroom announcement and word-of-mouth). Participants included undergraduate and graduate students between the ages of 18 and 35 years.

Additionally, participants were excluded if they: self-reported as a daily smoker,^14,15^ self-reported being pregnant,^16^ exercised within 5 hours of testing,^17^ consumed caffeine within 3 hours of testing,^18^ had a concussion or head trauma within the past 30 days,^19^ took marijuana or other illegal drugs within the past 30 days,^20^ or were considered a daily alcohol user (>30 drinks/month for women; >60 drinks/month for men).^21^

### Exercise protocol

The exercise bout involved exercising on a treadmill for 10 minutes. Participants exercised at 70% of their estimated heart rate max (220-age). This represents moderate-intensity exercise.^22^ The treadmill speed/incline was manipulated to keep the heart rate within 5 beats per minute of the target heart rate.

### Meditation protocol

For the meditation protocol, participants engaged in a 10-minute guided mindfulness meditation. Participants listened to this session from an audio-recorded tape with their eyes closed. The implemented guided meditation focused on breath/body present-moment awareness (through deep breathing exercises and a full-body scan), limiting mind-wandering/letting go of distractions or worries (i.e., enhanced attentional control), and cultivating relaxation. A Yoga Alliance 200-hour registered yoga teacher recorded the meditation session on the tape.

### Control protocol

During the control and seated conditions, participants completed a medium-level, on-line administered, Sudoku puzzle. The website for this puzzle is located here: https://www.websudoku.com/. We have experimental evidence that this control scenario does not prime or enhance memory function.^23^

### Learning and memory assessment

Learning and long-term memory (retrospective memory) were assessed using the standardized Rey Auditory Verbal Learning Test (RAVLT).^24^ Participants were asked to listen to and immediately recall a recording of a list of 15 words (List A) five times in a row (Trials 1-5). Each word list was recorded at a rate of approximately 1 word per second. Participants listened to and immediately recalled a list of 15 new words (List B). Immediately following the recall of List B, participants were asked to recall the words from List A (Trial 6). The average recall performance across the 6 trials was scored as a learning index. After a 10-minute delay, participants recalled as many words as possible from List A, constituting the long-term memory assessment. Separate word lists, of equal difficulty, were used for each visit.

### Statistical analysis

All statistical analyses were computed in JASP (v. 0.10.0). A one-factor repeated-measures ANOVA was computed for two memory outcomes, including the learning outcome (average performance across the 6 trials) and the long-term memory recall (10-min delay). In the ANOVA model, if the sphericity assumption was violated, we reported the Huynh-Feldt corrected values. Statistical significance was set at an alpha of 0.05. Partial eta-squared (η^2^_p_) was calculated as an effect size estimate for the ANOVA models.

## Results

### Demographic characteristics

Table 2 displays the characteristics of the sample. The sample, on average, was 21.6 (0.7) years, with gender being equally distributed (50% female).

### Physiological characteristics

Table 3 displays the physiological (heart rate) data across the exercise conditions. There was a significant main effect for time, *F*(3, 57) = 354.7, *P* = 0.001, η^2^_p_ = 0.95, but not main effect for condition,*F*(1, 19) = 0.002, *P* = 0.96, η^2^_p_ = 0.001, or time by condition interaction, *F*(3, 57) = 0.21, *P* = 0.89, η^2^_p_ = 0.01.

### Learning and memory results

**Figure 1:**
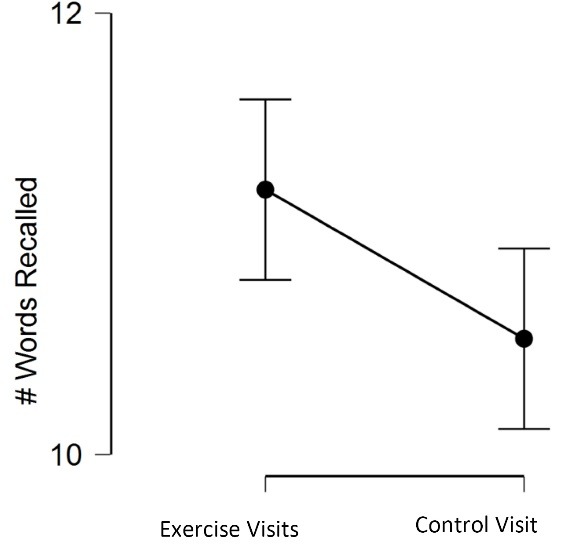
Learning effects (average across 6 learning trials) for the exercise visits vs. control visit. Error bars represent 95% CI.

Table 4 displays the memory scores across the experimental conditions. For the learning outcome, there was a significant main effect for learning, *F*(1.66, 31.54) = 6.05, *P* = 0.009, η^2^_p_ = 0.24. Bonferroni-corrected post hoc tests indicated that learning was not different between the Exercise Only and Exercise + Meditation visits, *M*_diff_ = -0.46 (95% CI: -0.85, -0.08, *P* = 0.07, or between the Exercise Only and Control visits, *M*_diff_ = 0.44 (95% CI: -0.11, 1.00), *P* = 0.34. However, Exercise + Meditation had a significantly greater learning effect than Control, *M*_diff_ = 0.90 (95% CI: 0.25, 1.57), *P* = 0.02. When collapsing the two exercise conditions together (Exercise Only and Exercise + Meditation), the exercise conditions had a greater learning effect when compared to the Control visit, *M*_diff_ = 0.68 (95% CI: 0.10, 1.25), *P* = 0.02 (Figure 1).

**Figure 2:**
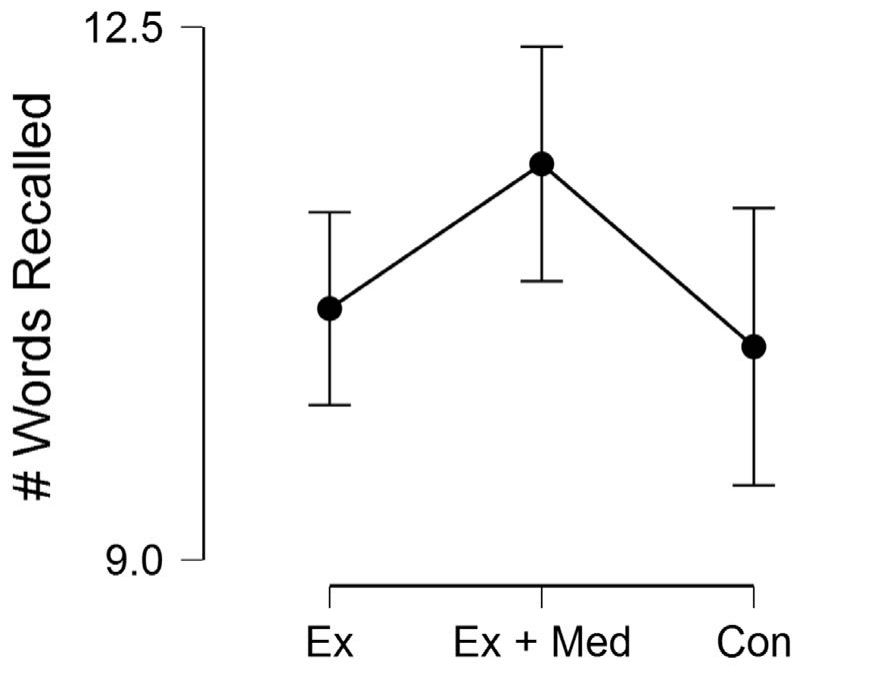
Long-term memory performance (10-min delay) across the three experimental conditions. Ex, Exercise; Med, Meditation; Con, Control. Error bars represent 95% CI.

For the long-term memory outcome (Figure 2), there was a marginally significant main effect for condition, *F*(2, 38) = 2.89, *P* = 0.06, η^2^_p_ = 0.13. The Exercise + Memory visit had better long-term memory when compared to Exercise Only, *M*_diff_ = 0.95 (95% CI: 0.07, 1.83), *P* = 0.03. Similarly, the Exercise + Memory visit had a marginally better long-term memory when compared to the Control visit, *M*_diff_ = 1.20 (95% CI: -0.07, 2.48), *P* = 0.06. The Exercise Only visit did not differ in long-term when compared to the control visit, *M*_diff_ = 0.25 (95% CI: -0.86, 1.36), *P* = 0.64.

## Discussion

The present experiment was designed to evaluate whether acute exercise could enhance learning and memory, and whether acute exercise coupled with mindfulness meditation, had a superior effect in long-term memory function when compared to exercise alone. The main findings of this experiment are twofold, including suggestive evidence that acute exercise enhanced learning, and when coupling acute exercise prior to encoding and meditation during early consolidation, long-term memory was enhanced.

Our suggestive findings of an acute exercise-induced learning effect align with past experimental studies.^25^ A speculated mechanism of this effect includes exercise-induced increases in neuronal excitability in key memory-related brain structures.^26^ This may help to facilitate long-term potentiation of the engram associated with the learning stimuli.^10^ We have discussed this synaptic tagging and capturing effect in detail elsewhere.^26^

Meditation during the consolidation stage may help to facilitate the replay of neurons that are part of the engram. For example, acute meditation has been shown to activate neural networks associated with memory retrieval.^27^ Further, meditation has been shown to facilitate memory consolidation in a motor memory paradigm.^13^ It would be interesting for future work to re-evaluate this experimental paradigm and consider other temporal periods of acute exercise and meditation on learning and memory. For example, other research demonstrates that an acute meditation session may optimize neural activity during a memory task,^28^ and as such, it would be worthwhile to implement the meditation session shortly before memory encoding. In addition to acute exercise, future work should consider the combined effects of chronic exercise and chronic meditation training on memory function.^29^ Such work should aim to overcome the limitations of the present experiment, including population homogeneity.

In conclusion, the present experiment provides suggestive evidence that acute exercise may enhance learning and, when coupling acute exercise prior to encoding and meditation during early consolidation, long-term memory may be enhanced. Future mechanistic work is needed to evaluate potential mechanisms of this effect and whether these two behaviors (exercise and meditation) induce unique or similar mechanisms that induce a synergistic effect on memory. Future work should aim to address the limitations of this study. For example, future work should include a larger, more representative sample and evaluate multiple exercise intensities.

## Ethical approval

This study was approved by the ethics committee at the University of Mississippi (#18-115, approved on May 30, 2018). Participants provided written consent prior to participation.

## Competing interests

The authors declare that they have no competing interests.

## Funding

No funding was used to prepare this manuscript.

## Authors’ contributions

MA collected the data and provided feedback and approval of the final manuscript. PL conceptualized the study, analyzed the data and prepared the initial draft of the manuscript.

**Table 1:** Study protocol across the three within-subject, counterbalanced visits

**Condition**	**Start – – – –**	**– – – – – **	**– – – – – **	**– – – – – **	**– – → Finish**
Exercise only	10-min of exercise	5-min seated rest (Sudoku)	Learning Task	10-min seated rest (Sudoku)	Delayed memory recall
Exercise and meditation	10-min of exercise	5-min seated rest (Sudoku)	Learning Task	10-min seated meditation	Delayed memory recall
Control	15-min seated rest (Sudoku)		Learning Task	10-min seated rest (Sudoku)	Delayed memory recall

**Table 2:** Participant characteristics across the sample (mean (SD))

**Variable**	**Point Estimate**	**SD**
Age, mean years	21.6	0.7
Gender, % female	50 (n=10)	-
Race-ethnicity, % White	95 (n=19)	-
BMI, mean kg/m^2^	23.8	3.5

**Table 3:** Heart rate data across the exercise visits (mean (SD))

**Heart Rate, mean bpm**	**Exercise Only**	**Exercise + Meditation**
Rest	74.9 (12.6)	76.1 (11.5)
Midpoint	121.1 (11.9)	121.6 (15.3)
Endpoint	133.1 (10.9)	131.2 (12.3)
Post (5 min)	83.6 (9.3)	83.4 (9.7)

**Table 4:** Learning and long-term memory scores across the experimental conditions (means (SD))

**Variable**	**Exercise Only**	**Exercise + Meditation**	**Control**
Learning index	10.96 (2.2)	11.43 (1.9)	10.52 (1.8)
Long-term memory	10.65 (3.5)	11.60 (3.4)	10.40 (3.1)
